# Supplementary material for: Antibiofilm Activity of Phorbaketals from the Marine Sponge Phorbas sp. against Staphylococcus aureus
Source: Mar Drugs. 2021 May 24;19(6):301. doi: 10.3390/md19060301 (PMC8225198; doi:10.3390/md19060301)
Supplement: Supplementary file 1 [file marinedrugs-19-00301-s001.zip › marinedrugs-1230727-supplementary.pdf]

Supplementary Materials

Antibiofilm activity of Phorbaketals from the Marine Sponge *Phorbas* sp. against *Staphylococcus aureus*

Contents

Characterization data	2
Figure S1. The ^{1}H NMR NMR spectrum of phorbaketal A acetate (**2**)	4
Figure S2. The ^{13}C NMR NMR spectrum of phorbaketal A acetate (**2**)	5
Figure S3. The COSY NMR spectrum of phorbaketal A acetate (**2**)	6
Figure S4. The HSQC NMR spectrum of phorbaketal A acetate (**2**)	7
Figure S5. The HMBC NMR spectrum of phorbaketal A acetate (**2**)	8
Figure S6. The ROESY NMR spectrum of phorbaketal A acetate (**2**)	9
Figure S7. The HR-ESIMS spectreum of phorbaketal A acetate (**2**)	10
Figure S8. The ^{1}H NMR NMR spectrum of phorbaketal B acetate (**4**)	11
Figure S9. The ^{13}C NMR NMR spectrum of phorbaketal B acetate (**4**)	12
Figure S10. The COSY NMR spectrum of phorbaketal B acetate (**4**)	13
Figure S11. The HSQC NMR spectrum of phorbaketal B acetate (**4**)	14
Figure S12. The HMBC NMR spectrum of phorbaketal B acetate (**4**)	15
Figure S13. The ROESY NMR spectrum of phorbaketal B acetate (**4**)	16
Figure S14. The HR-ESIMS spectreum of phorbaketal B acetate (**4**)	17
Figure S15. The ^{1}H NMR NMR spectrum of phorbaketal C acetate (**6**)	18
Figure S16. The ^{13}C NMR NMR spectrum of phorbaketal C acetate (**6**)	19
Figure S17. The COSY NMR spectrum of phorbaketal C acetate (**6**)	20
Figure S18. The HSQC NMR spectrum of phorbaketal C acetate (**6**)	21
Figure S19. The HMBC NMR spectrum of phorbaketal C acetate (**6**)	22
Figure S20. The HR-ESIMS spectreum of phorbaketal C acetate (**6**)	23
Figure S21. Planktonic cell growth in the presence of phorbaketals	24
Table S1. ^{1}H and ^{13}C NMR data of **2**, **4** and **6**	25
Table S2. Sequences of the primers used for qRT-PCR	26

Characterization data

Phorbaketal A (1). Yellow oil. $[\alpha]^{25}_{D}$ -118.1 (*c* 0.15, MeOH). IR (film) ν_{max}: 3434, 2914, 1681, 983 cm^{-1}. ^1H NMR (500 MHz, methanol-*d*4) δ 6.68 (dd, 1H, *J* = 5.5, 1.2 Hz, H-2), 5.54 (br s, 1H, H-10), 5.28 (br s, 1H, H-12), 5.22 (br s, 1H, H-17), 5.12 (br t, 1H, H-22), 4.74 (ddd, 1H, *J* = 11.3, 8.1, 3.4 Hz, H-16), 4.49 (dd, 1H, *J* = 5.5, 3.3 Hz, H-1), 4.07 (d, 1H, *J* = 14.7 Hz, H-9b), 4.03 (d, 1H, *J* = 14.7 Hz, H-9a), 2.59 (ddd, 1H, *J* = 13.7, 3.9, 3.4, H-7), 2.58 (dd, 1H, *J* = 16.1, 3.9 Hz, H-6b), 2.43 (dd, 1H, *J* = 16.1, 13.7 Hz, H-6a), 2.13 (m, 2H, H-21), 2.05 (m, 2H, H-20), 1.81 (s, 3H, H-4), 1.77 (s, 3H, H-19), 1.75 (s, 3H, H-14), 1.68 (s, 3H, H-24), 1.61 (s, 3H, H-25); ^{13}C NMR (125 MHz, methanol-*d*4) δ 202.7 (C-5), 143.7 (C-8), 142.0 (C-18), 141.6 (C-2), 139.5 (C-3), 138.7 (C-13), 132.6 (C-23), 125.8 (C-17), 125.0 (C-22), 124.7 (C-10), 123.0 (C-12), 96.1 (C-11), 66.9 (C-16), 64.7 (C-1), 63.8 (C-9), 40.6 (C-20), 38.8 (C-6), 36.4 (C-15), 34.7 (C-7), 27.5 (C-21), 25.9 (C-24), 22.8 (C-14), 17.8 (C-25), 16.9 (C-19), 15.9 (C-4); $[\alpha]^{25}_{D}$ -118.2 (*c* 0.15, MeOH); HRESIMS (positive-ion mode) *m*/*z*: 399.2535 $[M + H]^+$ (Calcd for $C_{25}H_{35}O_4$, 399.2530).

9-Acethylphorbaketal A (2). Yellow oil. $[\alpha]^{25}_{D}$ -63.9 (*c* 0.15, MeOH). UV (MeOH) λ_{max} (log ε): 203 (4.4), 229 (3.9) nm. IR (film) ν_{max}: 2921, 1743, 1682, 1225, 987 cm^{-1}. ^1H (500 MHz) and ^{13}C (125 MHz) NMR data, see Table 1. HRESIMS (positive-ion mode) *m*/*z*: 463.2460 $[M + Na]^+$ (Calcd for $C_{27}H_{36}O_5Na$, 463.2455).

Phorbaketal B (3). Yellow oil. $[\alpha]^{25}_{D}$ -115.1 (*c* 0.1, MeOH). IR (film) ν_{max}: 3388, 2915, 1444, 975 cm^{-1}. ^1H NMR (500 MHz, methanol-*d*4) δ 5.52 (dq, 1H, *J* = 5.1, 1.6 Hz, H-2), 5.49 (dd, 1H, *J* = 1.5, 1.2 Hz, H-10), 5.23 (br s, 1H, H-12), 5.20 (dq, 1H, *J* = 8.2, 1.3 Hz, H-17), 5.11 (br t, 1H, *J* = 7.1 Hz, H-22), 4.72 (ddd, 1H, *J* = 11.3, 8.2, 3.3 Hz, H-16), 4.24 (m, 1H, H-1), 4.09 (dd, 1H, *J* = 13.9, 1.2 Hz, H-9b), 4.05 (dd, 1H, *J* = 13.9, 1.5 Hz, H-9a), 4.04 (br d, 1H, *J* =10.3 Hz, H-5), 2.08 (m, 1H, H-7), 2.06 (m, 1H, H-6b), 1.51 (ddd, 1H, *J* = 12.5, 11.5, 10.3 Hz, H-6a), 2.12 (m, 2H, H-21), 2.04 (m, 2H, H-20), 2.00 (dd, 1H, *J* = 17.2, 11.3 Hz, H-15a), 1.82 (dd, 1H, *J* = 17.2, 3.3 Hz, H-15b), 1.82 (s, 3H, H-4), 1.75 (s, 3H, H-19), 1.74 (s, 3H, H-14), 1.67 (s, 3H, H-24), 1.61 (s, 3H, H-25); ^{13}C NMR (125 MHz, methanol-*d*4) δ 145.7(C-3), 144.4(C-8), 142.1(C-18), 138.2(C-13), 132.5(C-23), 125.8(C-17), 125.0(C-22), 124.4(C-10), 123.7(C-12), 123.2(C-2), 95.6(C-11), 70.7(C-5), 66.6(C-16), 65.6(C-1), 63.9(C-9), 40.6(C-20), 36.4(C-15), 34.4(C-7), 33.0(C-6), 27.5(C-21), 25.9(C-24), 22.9(C-14), 19.4(C-4), 17.8(C-25), 16.8(C-19); $[\alpha]^{25}_{D}$ -115.1 (c 0.1, MeOH); HRESIMS (positive-ion mode) m/z: 423.2501 $[M + Na]^+$ (Calcd for $C_{25}H_{36}O_4Na$, 423.2506).

5-Acethylphorbaketal B (4). Yellow oil. $[\alpha]^{25}_{D}$ -148.7 (*c* 0.1, MeOH). UV (MeOH) λ_{max} (log ε): 203 (4.4) nm. IR (film) ν_{max}: 2917, 1735, 1238, 980 cm^{-1}. ^1H (500 MHz) and ^{13}C (125 MHz) NMR data, see Table 1. HRESIMS (positive-ion mode) *m*/*z*: 465.2603 $[M + Na]^+$ (Calcd for $C_{25}H_{38}O_5Na$, 465.2611).

Phorbaketal C (5). Yellow oil. $[\alpha]^{25}_{D}$ -122.3 (*c* 0.1, MeOH). IR (film) ν_{max}: 3378, 2918, 1443, 985 cm^{-1}. ^1H NMR (500 MHz, methanol-*d*4) δ 5.58 (br d, 1H, *J* = 5.4 Hz, H-2), 5.51 (d, 1H, *J* = 1.2 Hz, H-10), 5.23 (br s, 1H, H-12), 5.21 (d, 1H, *J* = 8.3 Hz, H-17), 5.11 (br t, 1H, *J* = 7.1 Hz, H-22), 4.72 (ddd, 1H, *J* = 11.3, 8.3, 3.4 Hz, H-16), 4.28 (m, 1H, H-1), 4.08 (d, 2H, *J* = 1.2 Hz, H-9), 3.97 (dd, 1H, *J* = 3.7, 2.0 Hz, H-5), 2.32 (ddd, *J* = 13.2, 3.4, 3.2 Hz, 1H, H-7), 1.88 (ddd, 1H, *J* = 13.2, 3.2, 2.0 Hz, H-6b), 1.68 (ddd, 1H, *J* = 13.2, 13.2, 3.7 Hz, H-6a), 2.12 (m, 2H, H-21), 2.04 (m, 2H, H-20), 2.00 (dd, 1H, *J* = 17.4, 11.3 Hz, H-15a), 1.83 (dd, 1H, *J* = 17.2, 3.4 Hz, H-15b), 1.86 (s, 3H, H-4), 1.76 (s, 3H, H-19), 1.74 (s, 3H, H-14), 1.67 (s, 3H, H-24), 1.61 (s, 3H, H-25); ^{13}C NMR (125 MHz, methanol-*d*4) δ 144.6(C-8), 142.1(C-3), 138.2(C-13), 132.6(C-23), 125.8(C-17), 125.0(C-22), 124.3(C-2), 124.0(C-10), 123.7(C-12), 95.6(C-11), 68.5(C-5), 66.7(C-16), 65.3(C-1), 63.8(C-9), 40.6(C-20), 36.4(C-15), 32.7(C-6), 29.7(C-7), 27.5(C-21), 25.9(C-24), 22.8(C-14), 21.5(C-4), 17.8(C-25), 16.8(C-19); $[\alpha]^{25}_{D}$ -122.3 (c 0.10, MeOH); HRESIMS (positive-ion mode) m/z: 423.2503 $[M + Na]^+$ (Calcd for $C_{25}H_{36}O_4Na$, 423.2506).

5-Acethylphorbaketal C (6). Yellow oil. $[\alpha]^{25}_{D}$ -102.3 (*c* 0.1, MeOH). UV (MeOH) λ_{max} (log ε): 203 (4.4) nm. IR (film) ν_{max}: 2918, 1736, 1238, 977 cm^{-1}. ^{1}H (500 MHz) and ^{13}C (125 MHz) NMR data, see Table 1. HRESIMS (positive-ion mode) *m/z*: 465.2614 [M + Na]$^{+}$ (Calcd for $C_{25}H_{38}O_{4}Na$, 465.2611).

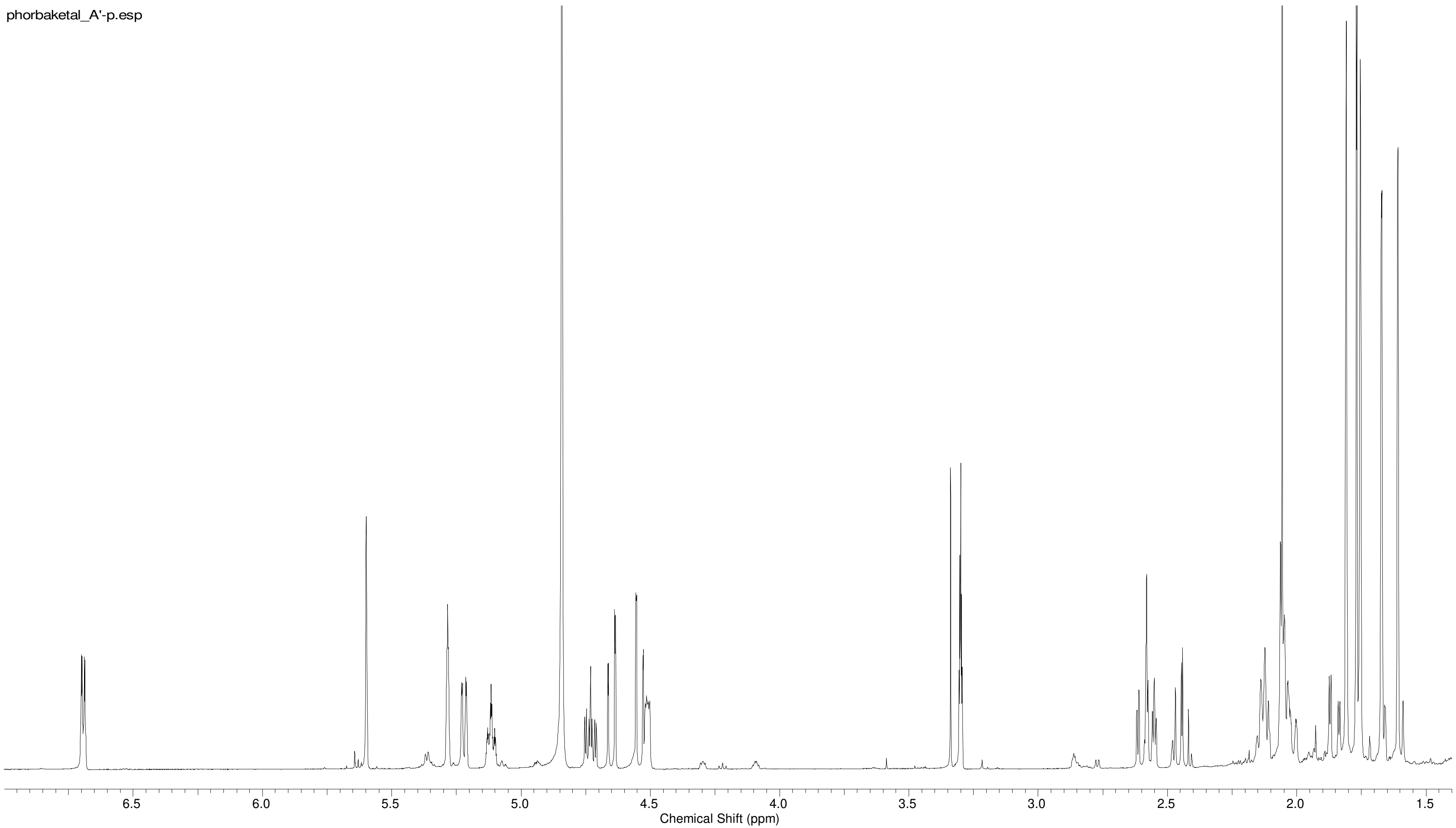

Figure S1. The ^{1}H NMR spectrum of phorbaketal A acetate (2)

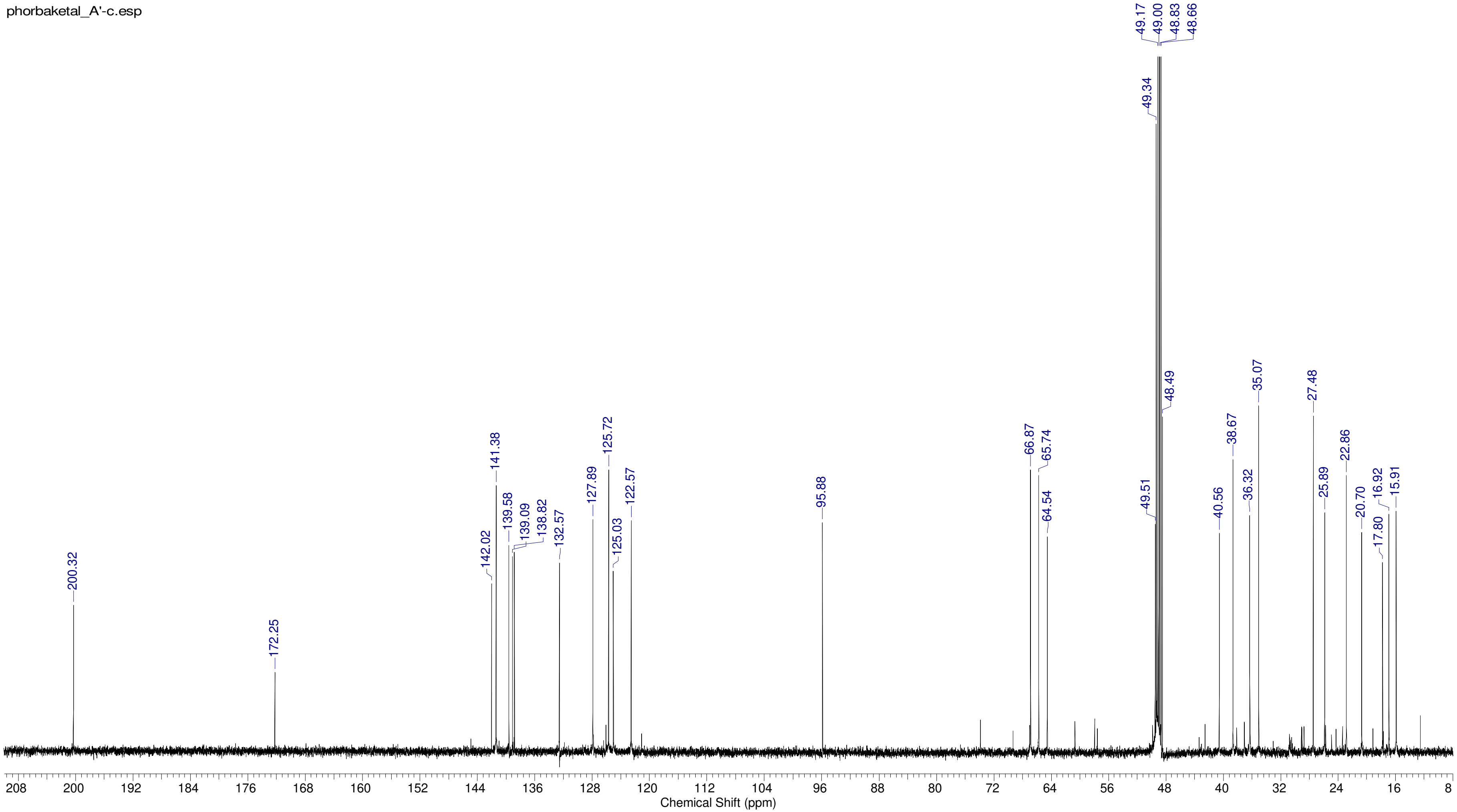

Figure S2. The ^{13}C NMR spectrum of phorbaketal A acetate (2)

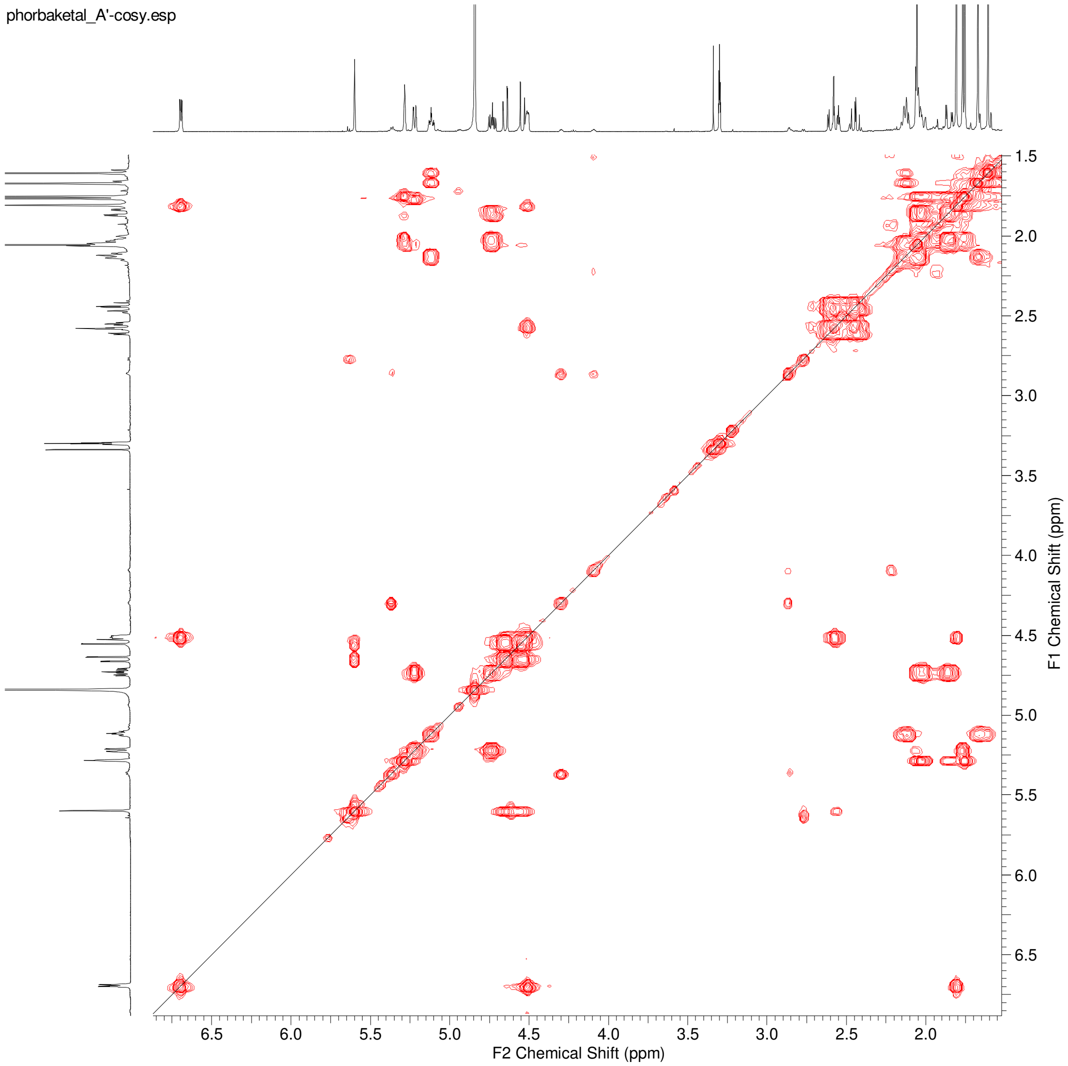

Figure S3. The COSY NMR spectrum of phorbaketal A acetate (2)

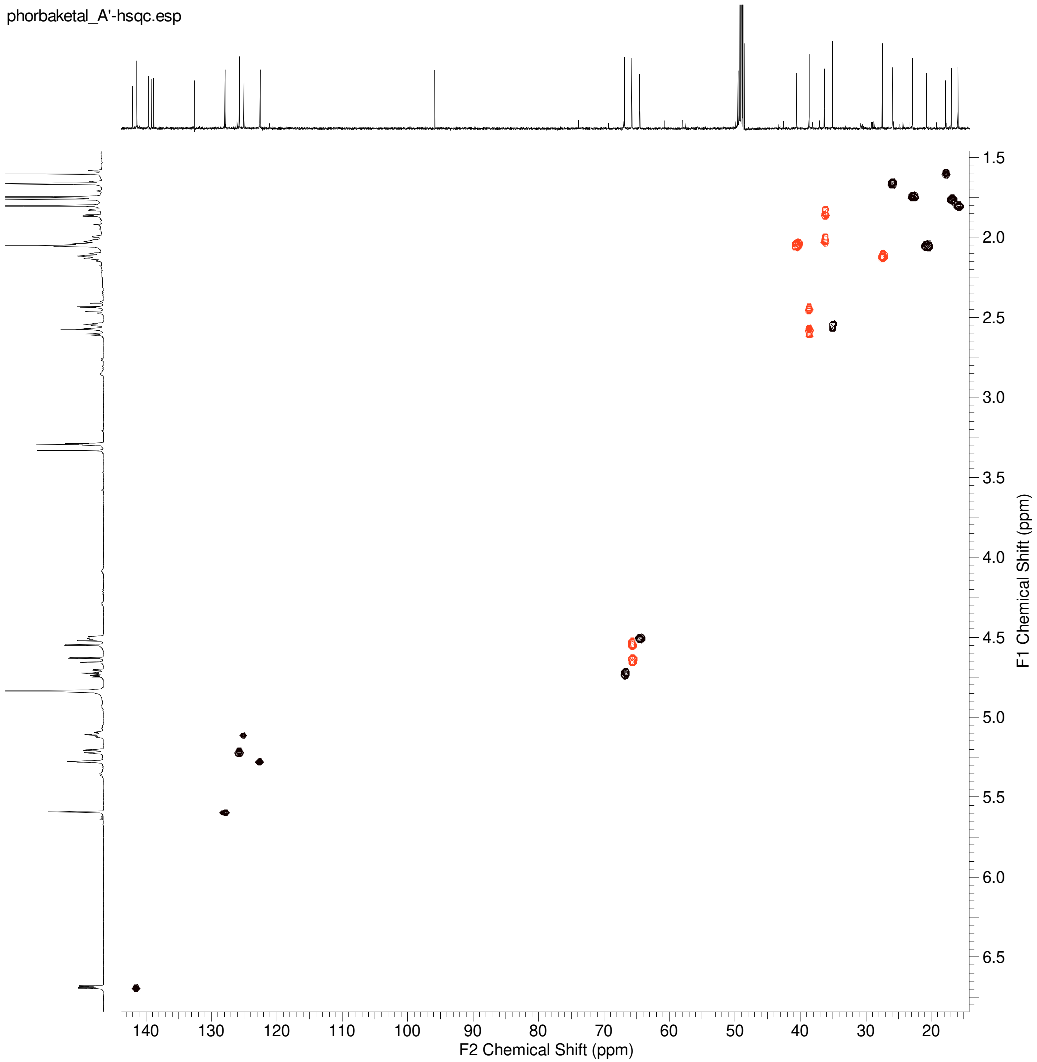

Figure S4. The HSQC NMR spectrum of phorbaketal A acetate (2) (red peaks: CH_2; black peaks: CH)

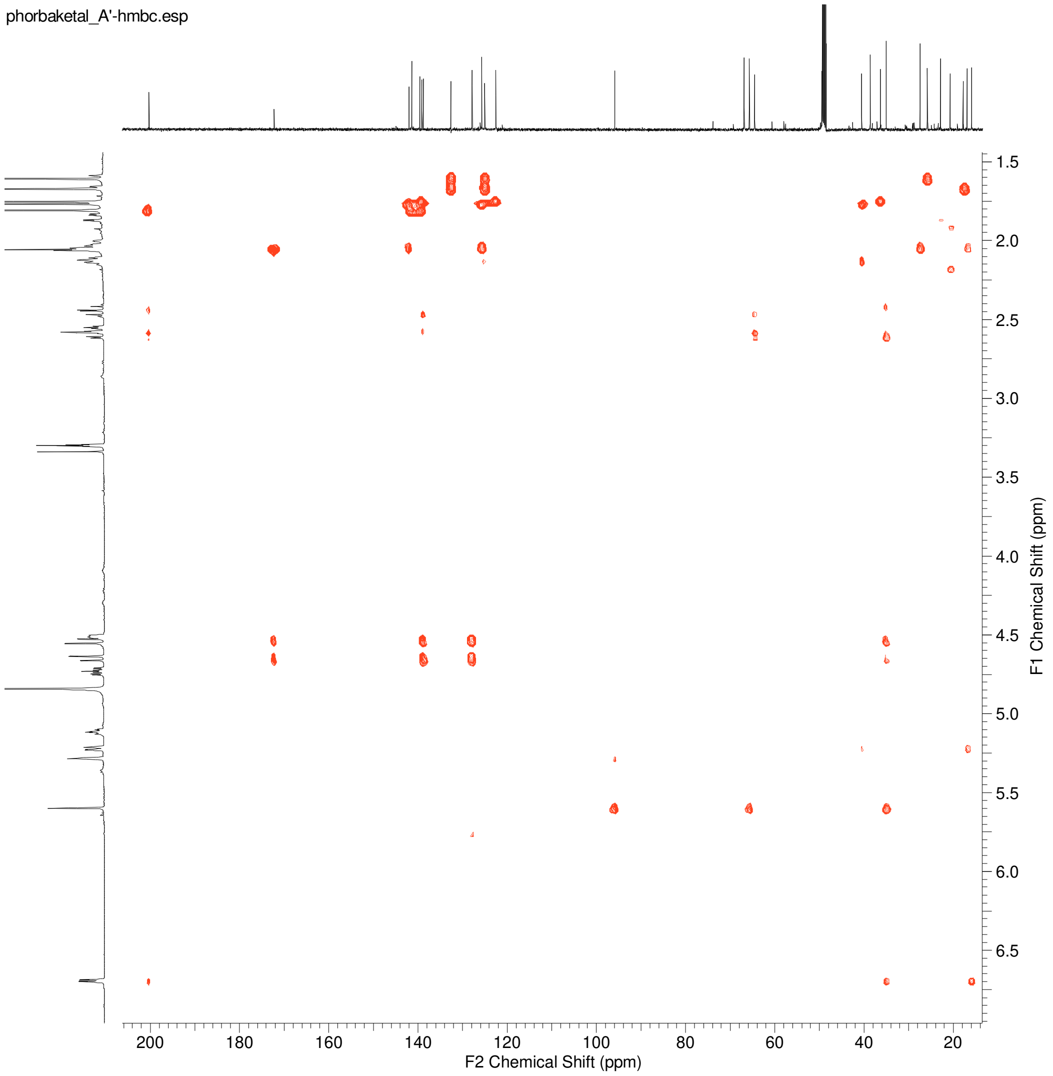

Figure S5. The HMBC NMR spectrum of phorbaketal A acetate (2)

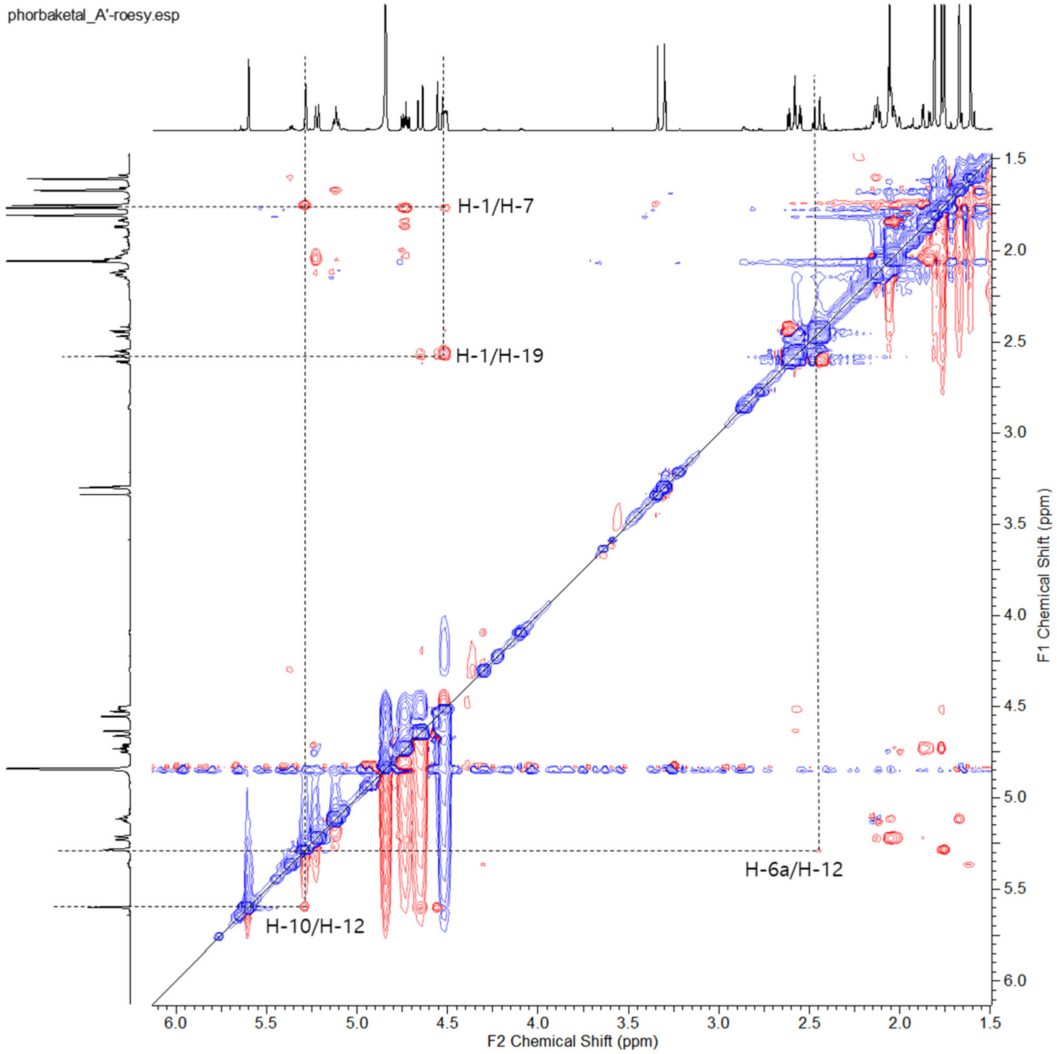

Figure S6. The ROESY NMR spectrum of phorbaketal A acetate (2)

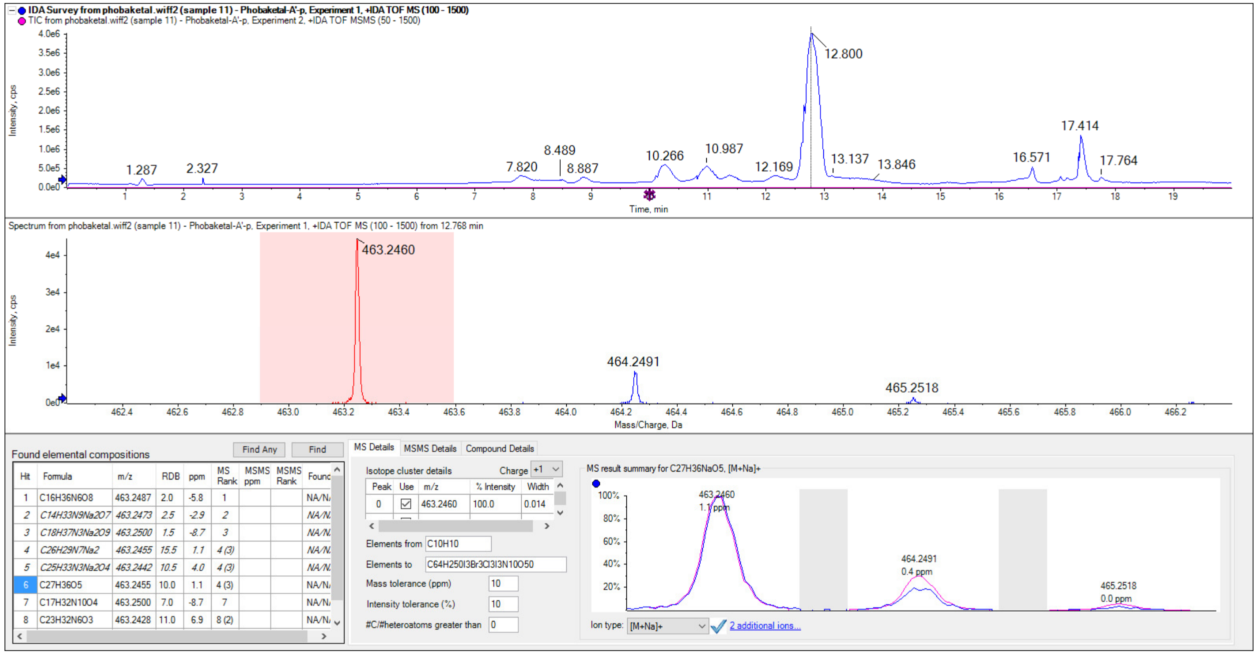

Figure S7. The HR-ESIMS of phorbaketal A acetate (2)

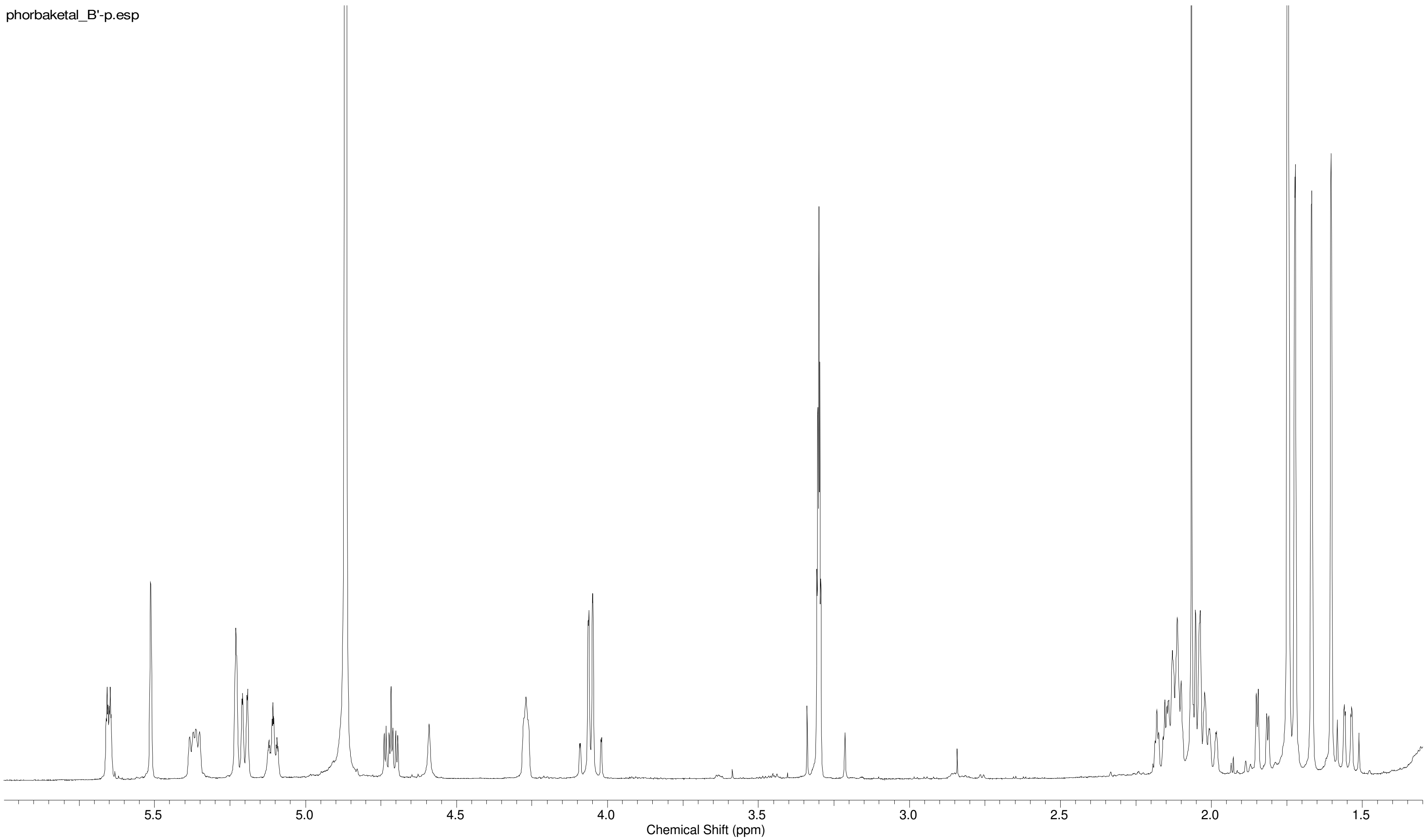

Figure S8. The ^{1}H NMR spectrum of phorbaketal B acetate (4)

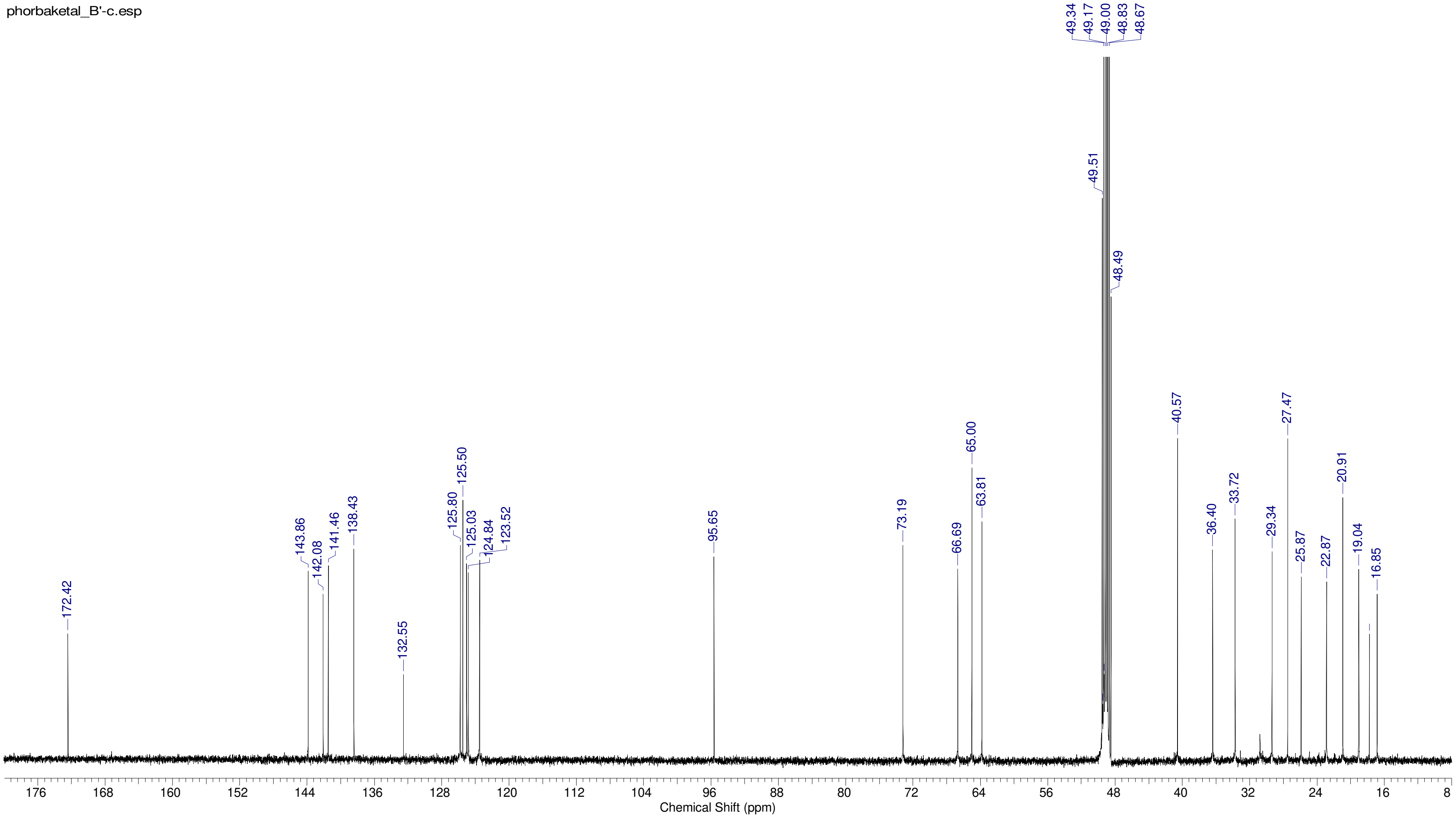

Figure S9. The ^{13}C NMR spectrum of phorbaketal B acetate (4)

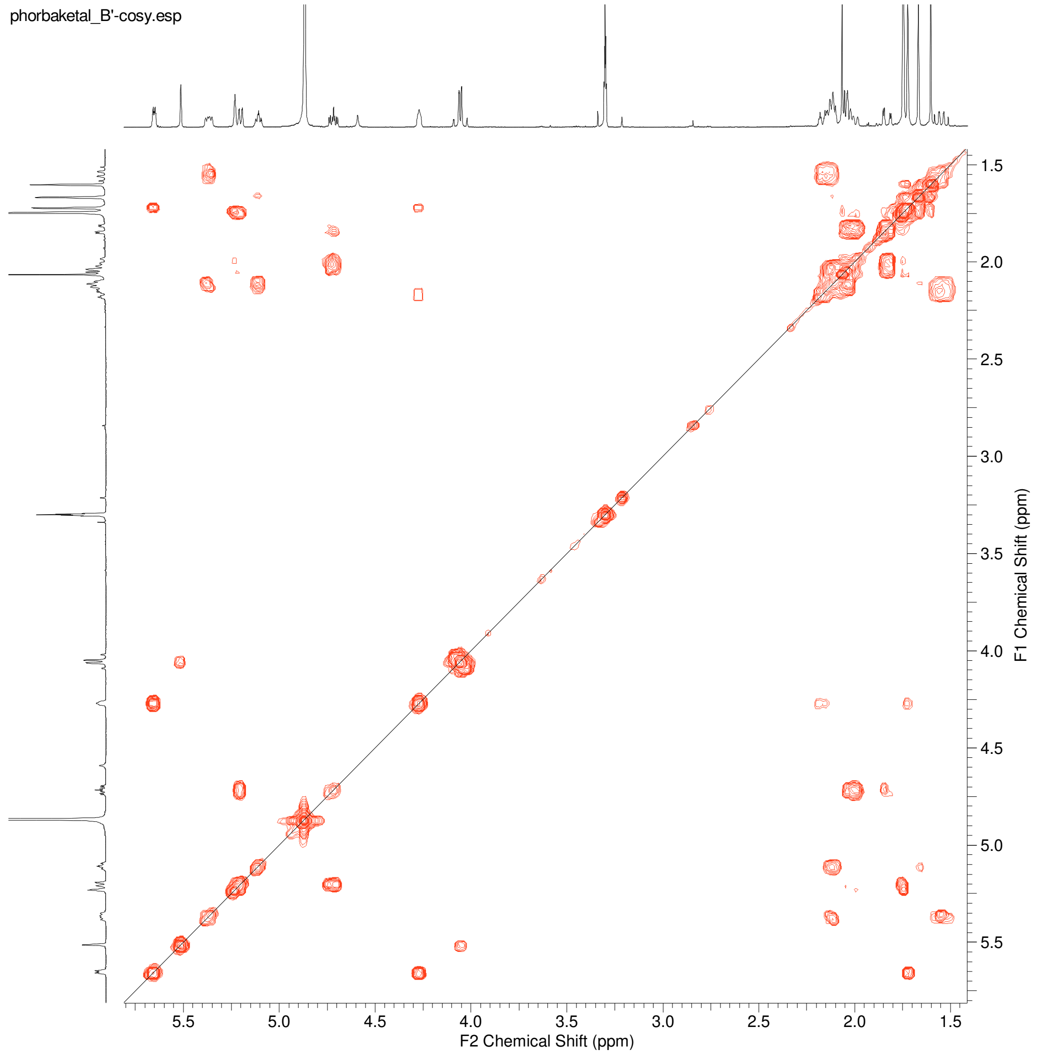

Figure S10. The COSY NMR spectrum of phorbaketal B acetate (4)

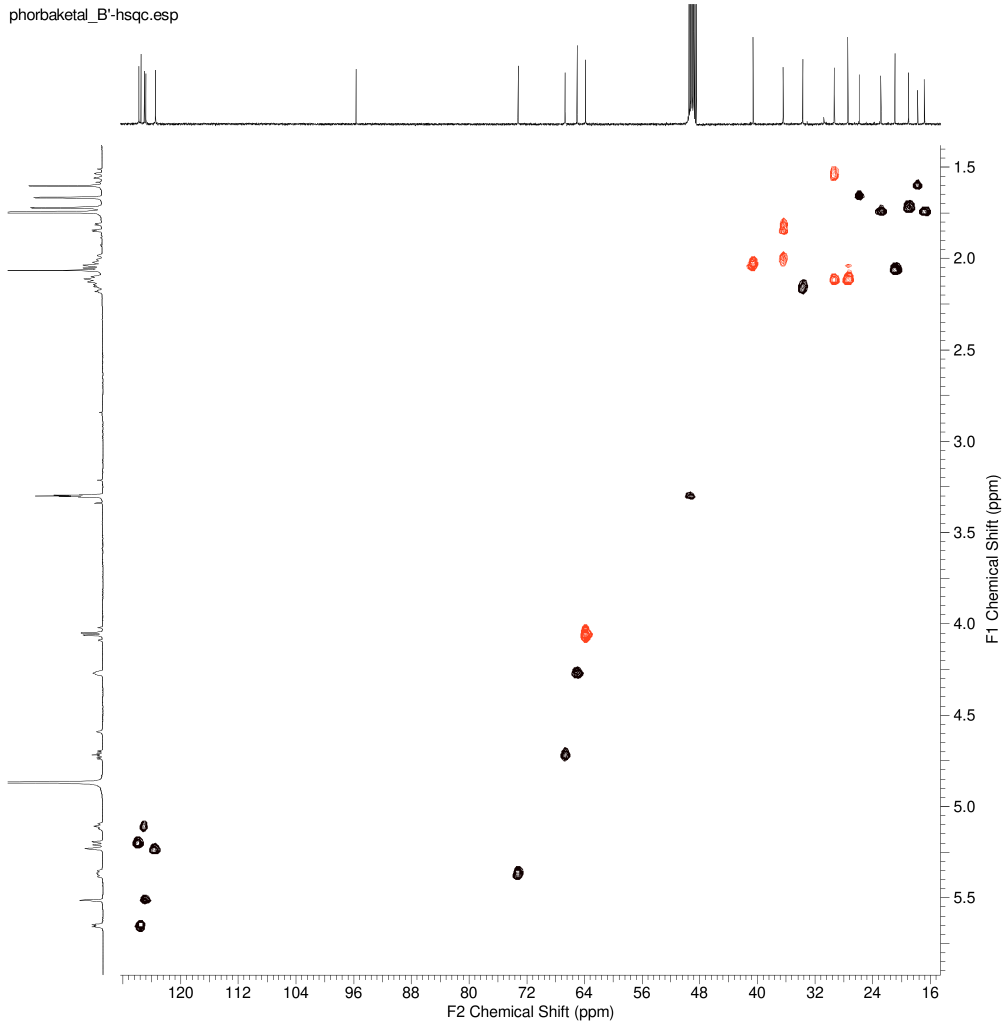

Figure S11. The HSQC NMR spectrum of phorbaketal B acetate (4) (red peaks: CH_2; black peaks: CH)

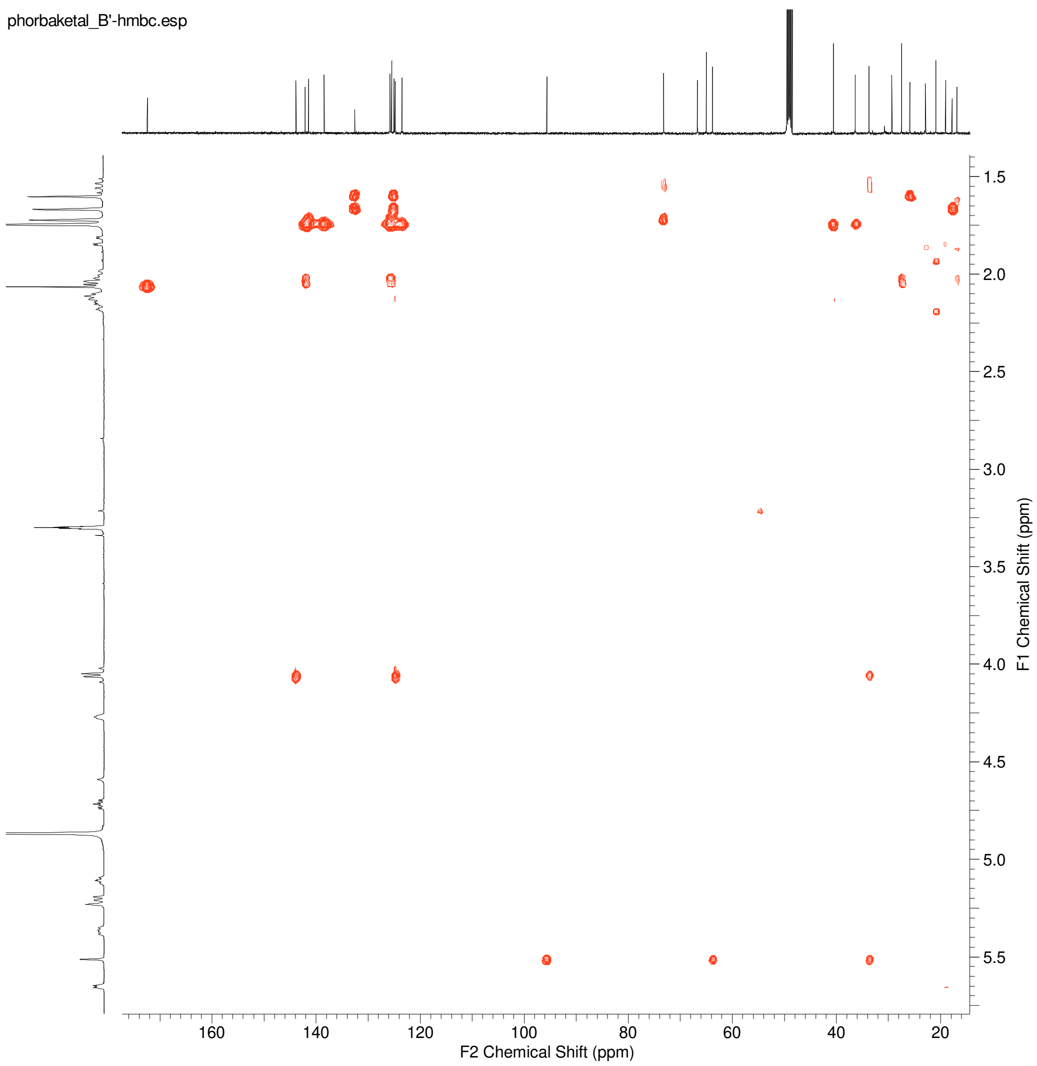

Figure S12. The HMBC NMR spectrum of phorbaketal B acetate (4)

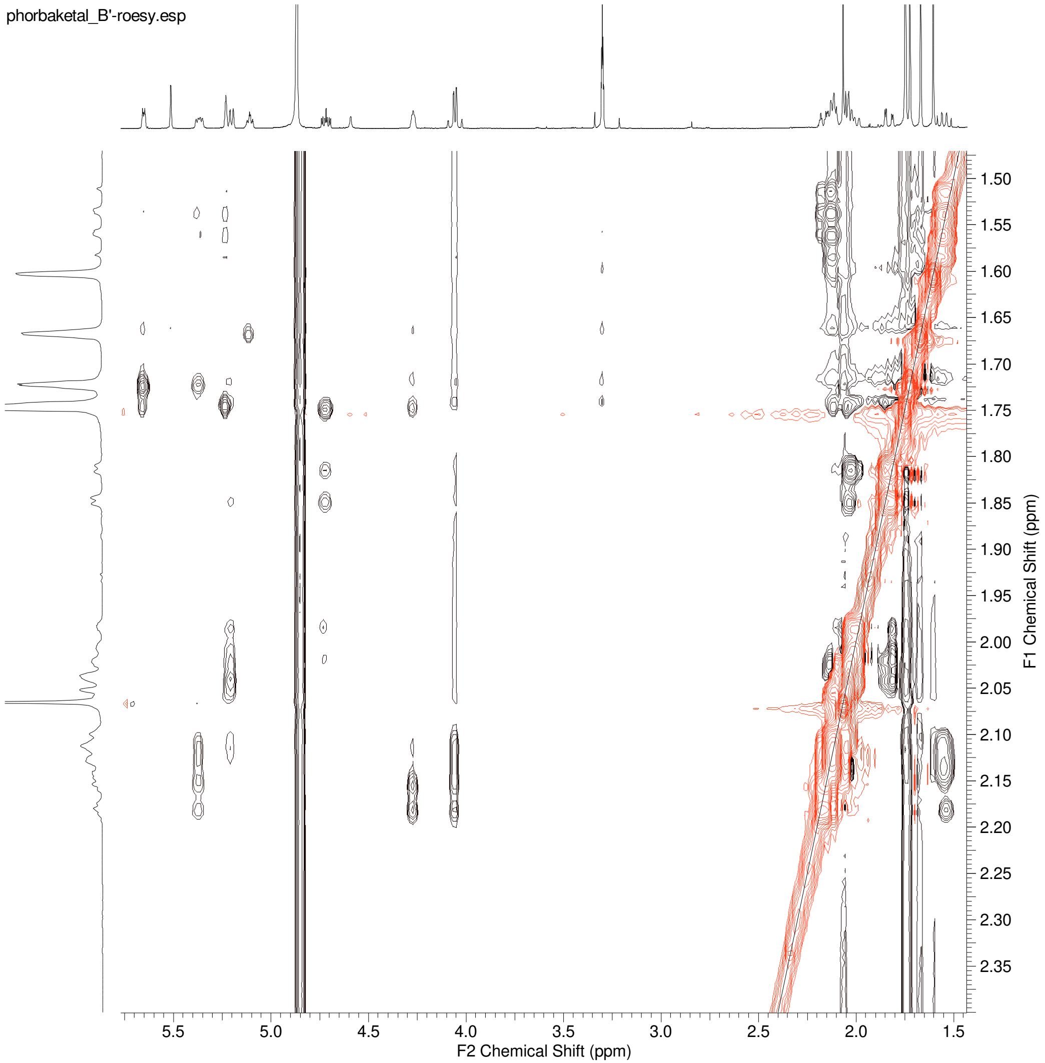

Figure S13. The ROESY NMR spectrum of phorbaketal B acetate (4)

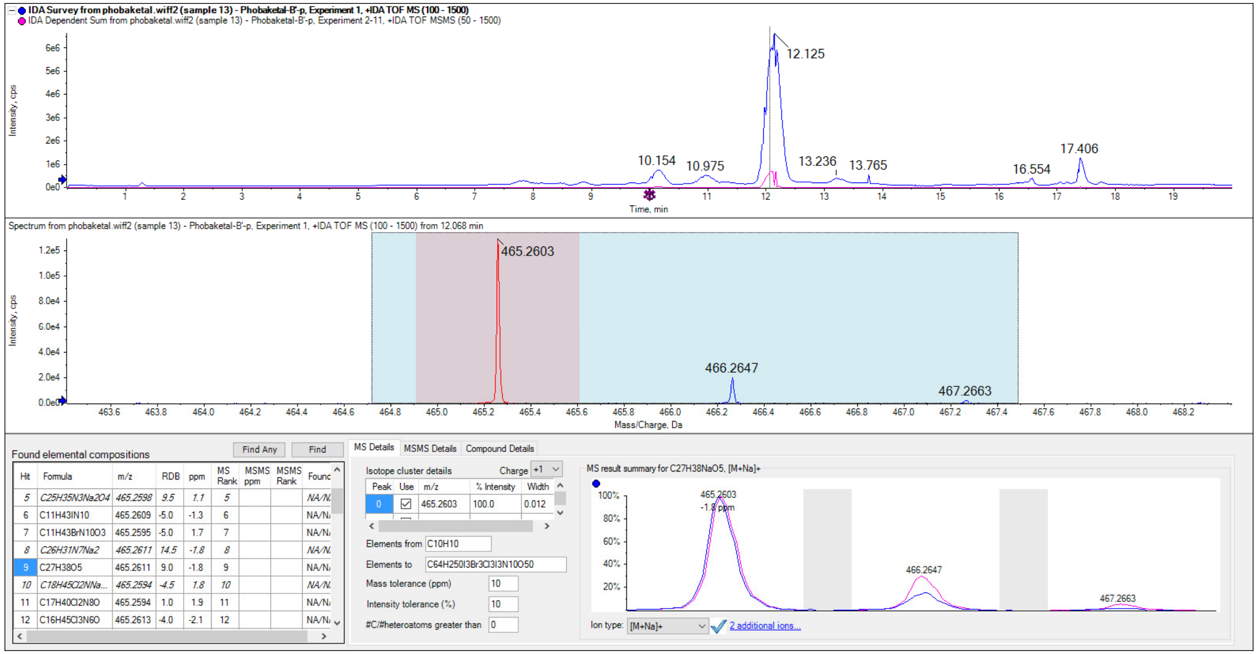

Figure S14. The HR-ESIMS of phorbaketal B acetate (4)

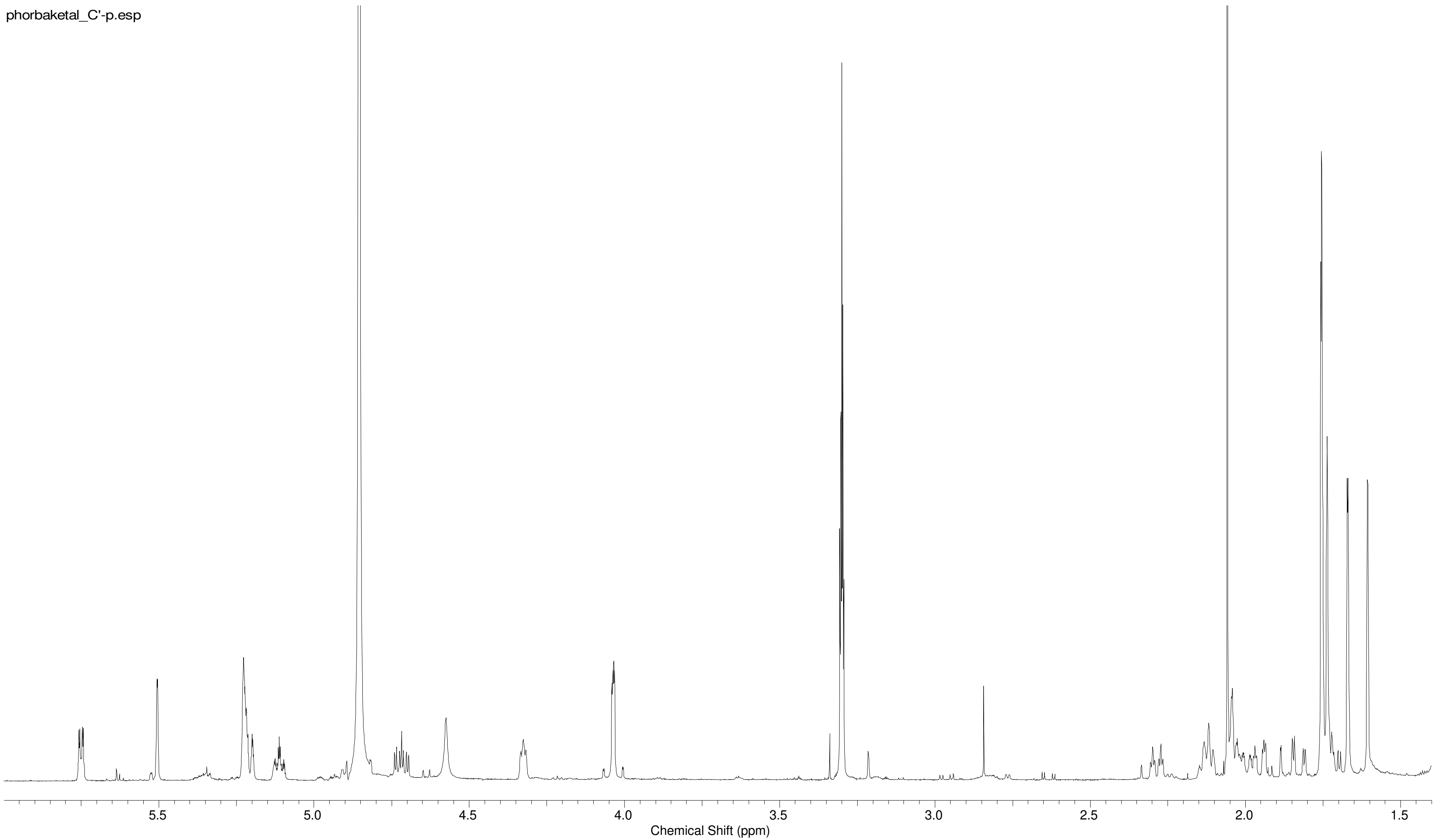

Figure S15. The ^{1}H NMR spectrum of phorbaketal C acetate (6)

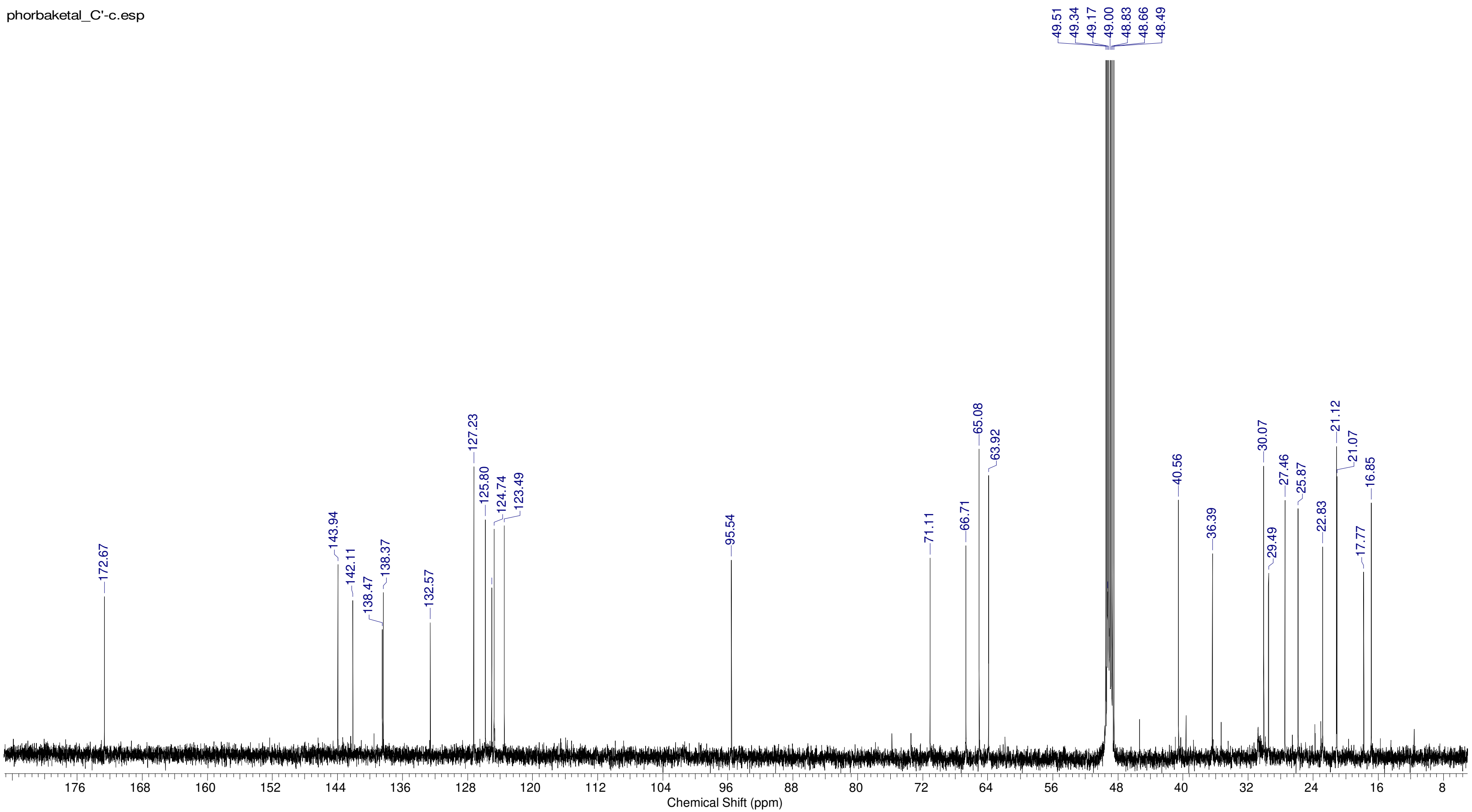

Figure S16. The ^{13}C NMR spectrum of phorbaketal C acetate (6)

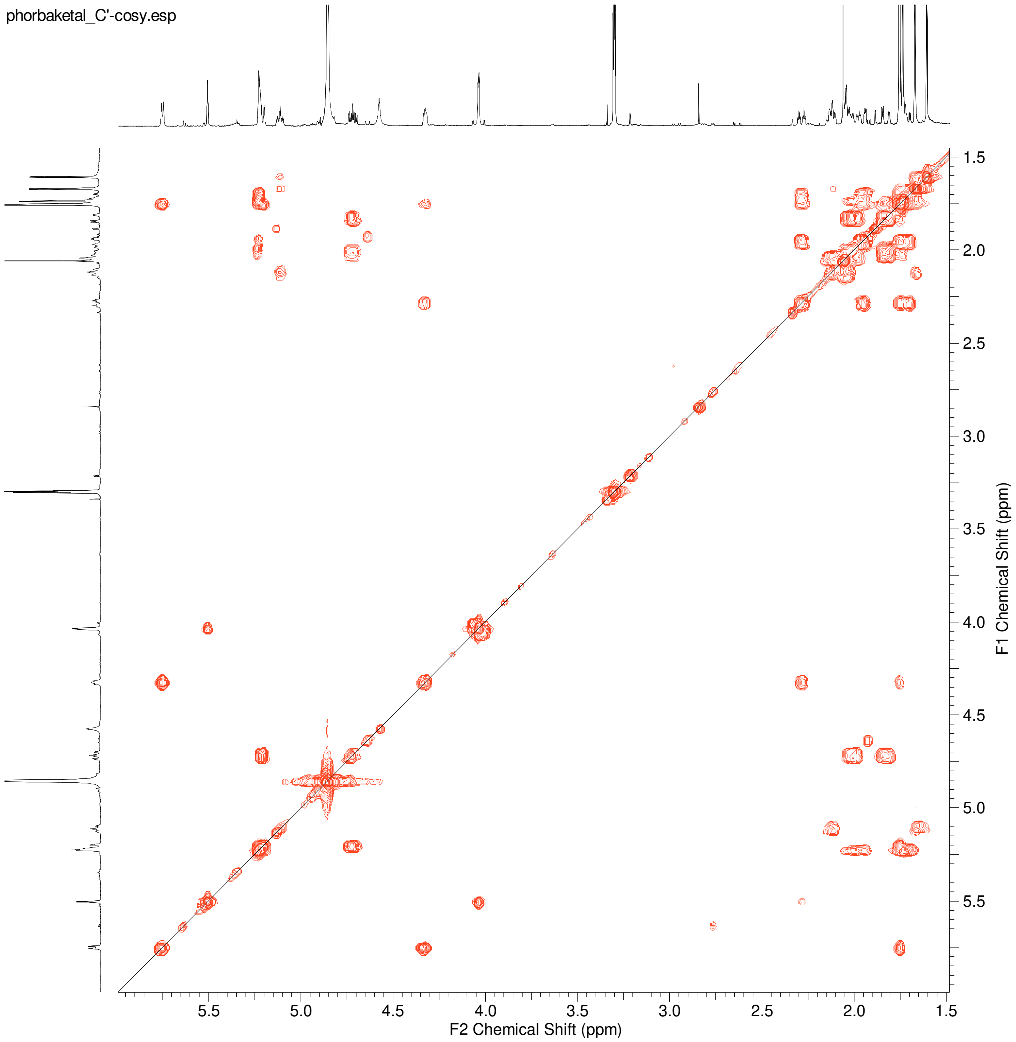

Figure S17. The COSY NMR spectrum of phorbaketal C acetate (6)

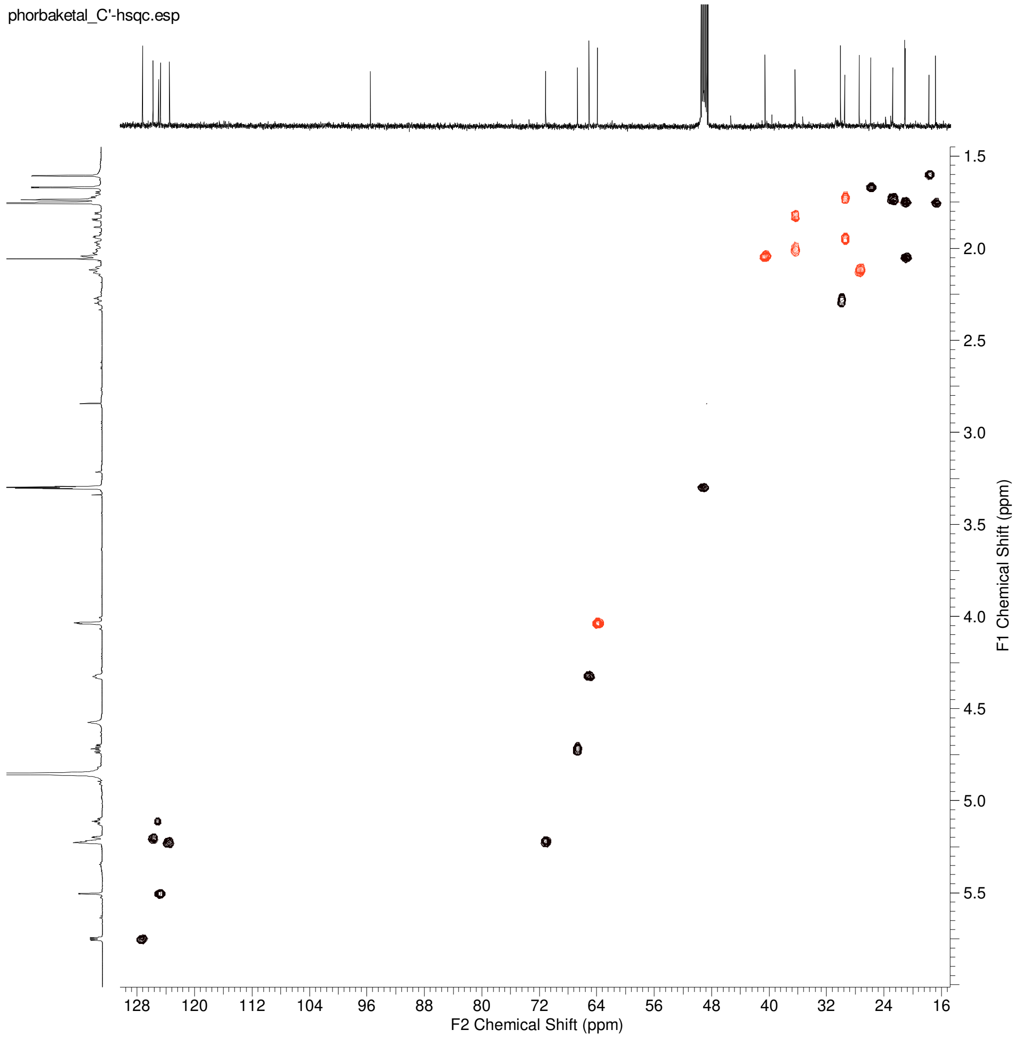

Figure S18. The HSQC NMR spectrum of phorbaketal C acetate (6) (red peaks: CH_2; black peaks: CH)

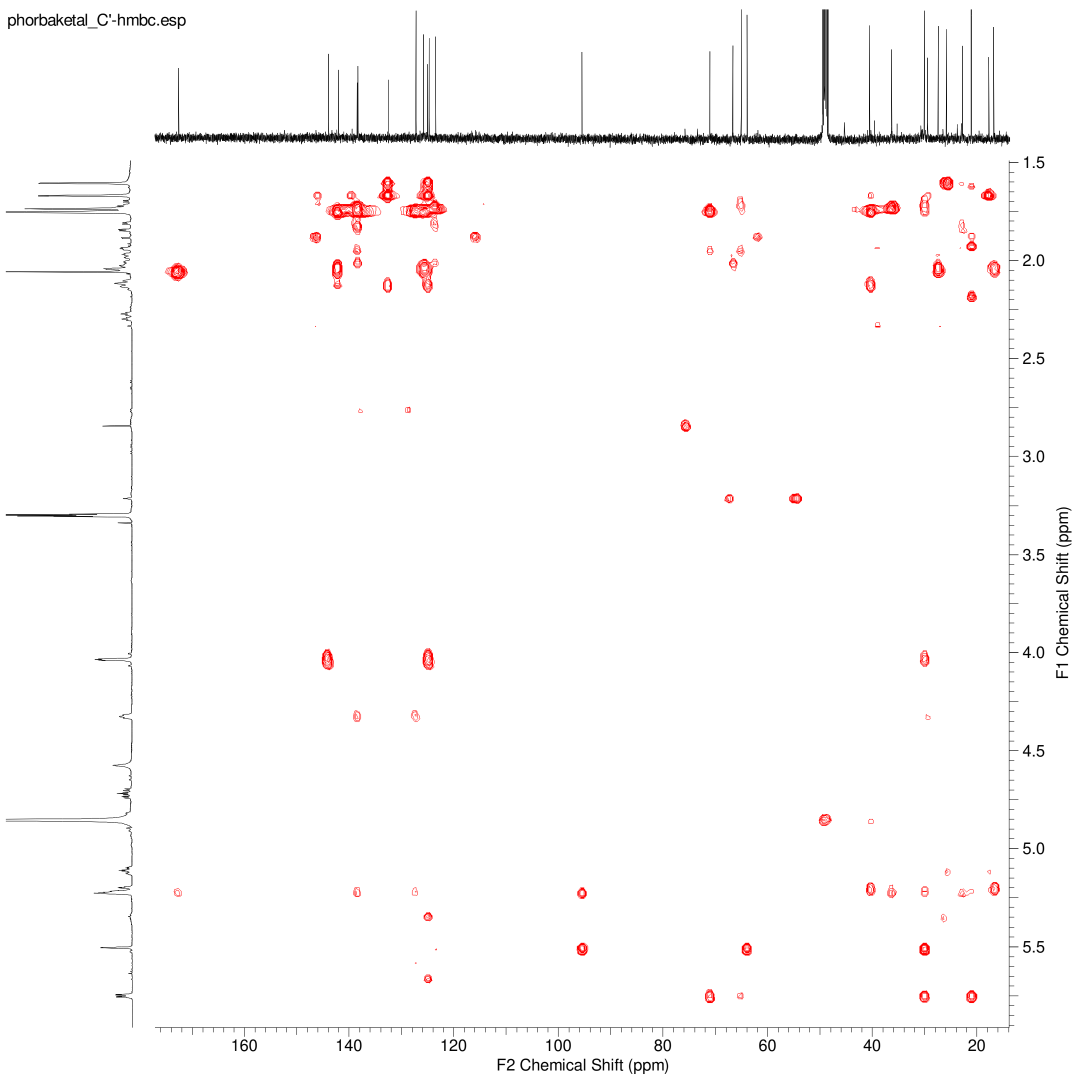

Figure S19. The HMBC NMR spectrum of phorbaketal C acetate (6)

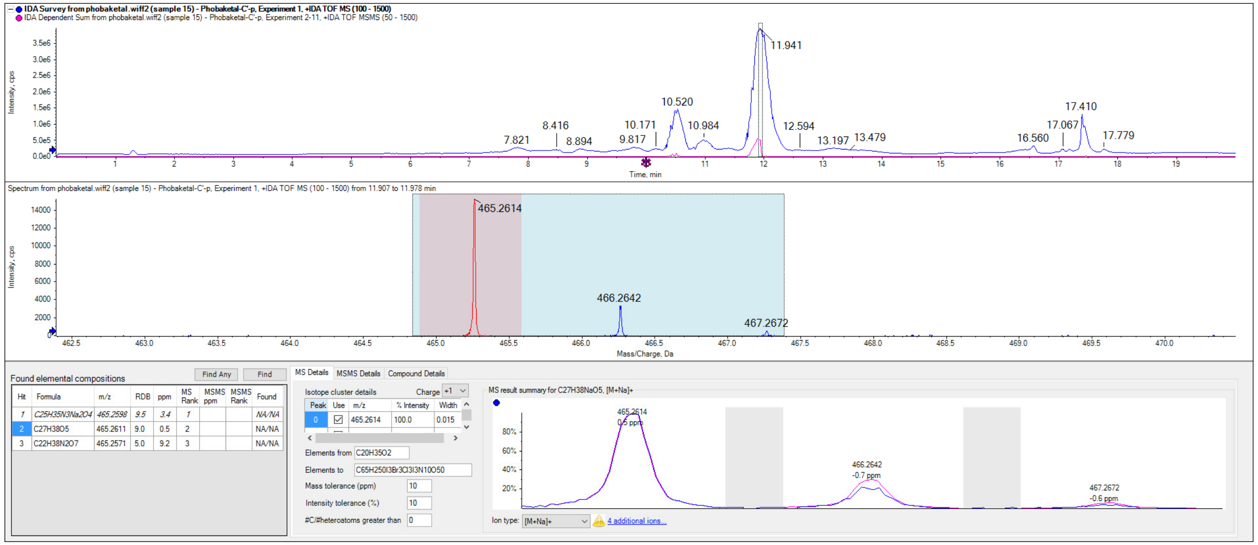

Figure S20. The HR-ESIMS of phorbaketal C acetate (6)

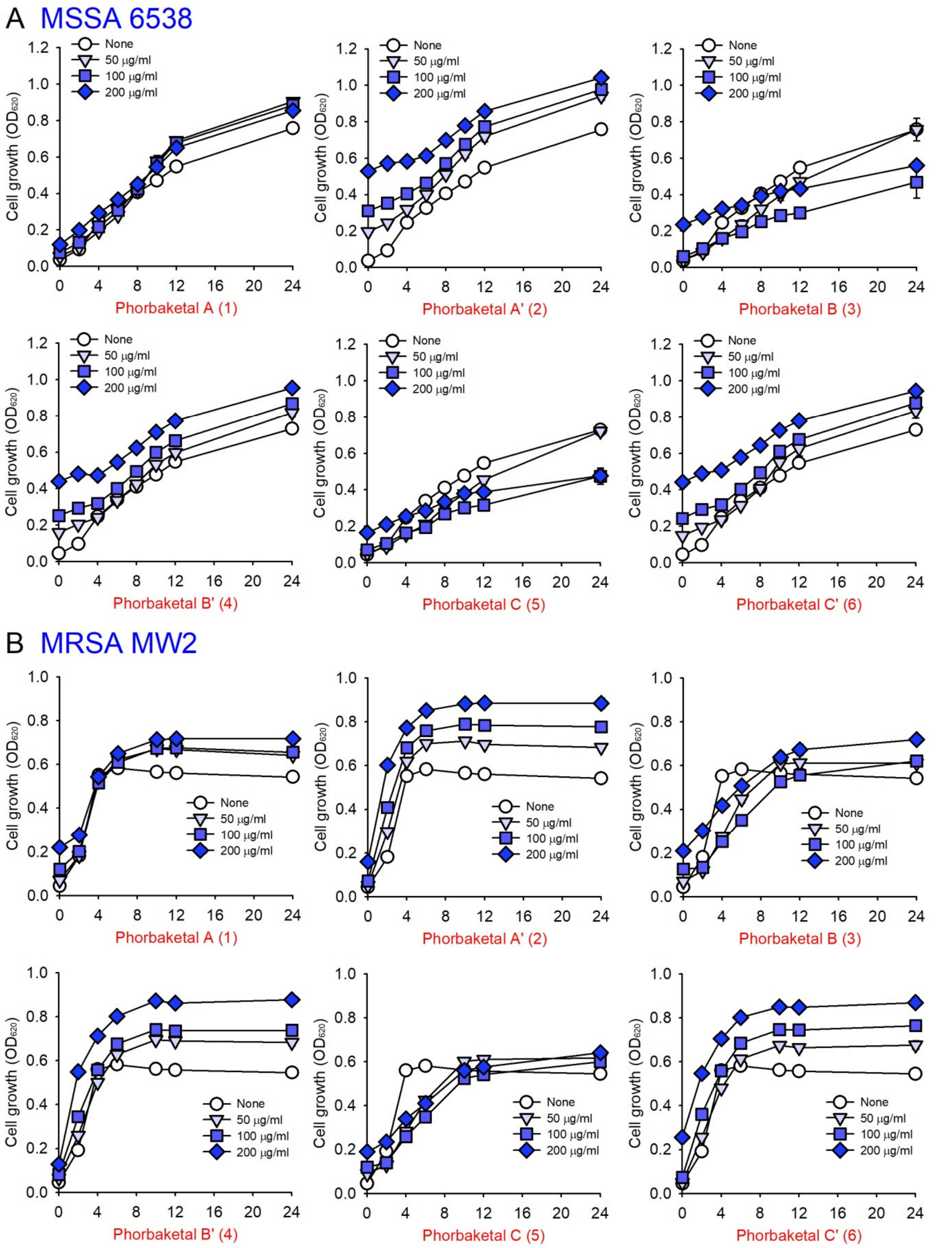

Figure S21. Planktonic cell growth in the presence of phorbaketals. Planktonic cell growths in the presence of different concentrations of phorbaketals were monitored in 96-well plates by measuring absorbance at 620 nm (OD_{620}) using a spectrophotometer (UV-160, Shimadzu, Japan). Two independent cultures were used.

Table S1. ^{1}H and ^{13}C NMR data of 2, 4 and 6 (500 and 125 MHz, respectively, methanol-*d*4)

	2			4		6	
no.	δ_C	δ_H (mult, *J* Hz)	HMBC	δ_C	δ_H (mult, *J* Hz)	δ_C	δ_H (mult, *J* Hz)
1	64.5, CH	4.51, m	C-2, 6, 11	65.0, CH	4.27, br t	65.1, CH	4.33, br t
2	141.4, CH	6.69, dq (5.8, 1.5)	C-1, 4, 5, 7	125.5, CH	5.65, dd (5.1, 1.6)	127.3, CH	5.75, dq (5.4, 1.5)
3	139.6, C			141.5, C		138.5, C	
4	15.9, CH_3	1.81, br s	C-2, 3, 5	19.0, CH_3	1.72, s	21.1, CH_3	1.75, s
5	200.3, C			73.2, CH	5.37, br q	71.1, CH	5.23, m
6	38.7, CH_2	a 2.44, dd (14.2, 11.7)	C-1, 5, 7, 8	29.3, CH_2	a 1.55, ddd (13.2, 11.7, 10.7)	29.5, CH_2	a 1.72, m
		b 2.59, m	C-1, 5, 7, 8		b 2.11, m		b 1.95, ddd (14.4, 4.0, 3.1)
7	35.1, CH	2.57, m	C-8	33.7, CH	2.17, dt (13.2, 2.9)	30.1, CH	2.28, dt (13.2, 3.1)
8	138.8, C			143.9, C		144.0, C	
9	65.7, CH_2	a 4.54, dd (14.3, 1.5)	C-7, 8, 10, 1'	63.8, CH_2	a 4.03, dd (14.2, 1.5)	63.9, CH_2	a 4.02, dd (14.1, 1.5)
		b 4.65, dd (14.3, 1.5)	C-7, 8, 10, 1'		b 4.08, dd (14.2, 1.5)		b 4.05, dd (14.1, 1.5)
10	127.9, CH	5.60, br s	C-7, 9, 11	124.8, CH	5.51, br d	124.7, CH	5.50, br q
11	95.9, C			95.7, C		95.5, C	
12	122.6, CH	5.28, m	C-11, 14, 15	123.5, CH	5.23, m	123.5, CH	5.23, m
13	139.1, C			138.4, C		138.3, C	
14	22.9, CH_3	1.75, s	C-12, 13, 15	22.9, CH_3	1.75, s	22.8, CH_3	1.74, s
15	36.3, CH_2	a 1.85, dd (17.4, 3.4)	C-12, 13	36.4, CH_2	a 1.83, dd (17.3, 3.3)	36.4, CH_2	a 1.83, br d (3.3)
		b 2.01, br d	C-12, 13		b 1.99, br d (11.1)		b 2.00, br d (11.0)
16	66.9, CH	4.73, ddd (11.3, 8.2, 3.4)	C-11, 17, 18	66.7, CH	4.72, ddd (11.1, 8.2, 3.3)	66.7, CH	4.75, ddd (11.0, 8.2, 3.3)
17	125.7, CH	5.22, dq (8.2, 1.2)	C-15, 19, 20	125.8, CH	5.20, dq (8.2, 1.2)	125.8, CH	5.21, br d (8.2)
18	142.0, C			142.1, C		142.1, C	
19	16.9, CH_3	1.77, s	C-17, 18, 20	16.9, CH_3	1.75, s	16.9, CH_3	1.76, s
20	40.6, CH_2	2.05, m	C-18	40.6, CH_2	2.05, m	40.6, CH_2	2.04, m
21	27.5, CH_2	2.12, m	C-20, 22, 23	27.5, CH_2	2.11, m	27.5, CH_2	2.12, m
22	125.0, CH	5.12, m	C-24, 25	125.0, CH	5.11, br t (7.0)	125.0, CH	5.11, br t (6.9)
23	132.6, C			132.6, C		132.6, C	
24	25.9, CH_3	1.67, s	C-22, 23, 25	25.9, CH_3	1.67, s	25.9, CH_3	1.67, s
25	17.8, CH_3	1.61, s	C-22, 23, 24	17.8, CH_3	1.61, s	17.8, CH_3	1.61, s
O-Ac							
1'	172.3, C			172.4, C		172.6, C	
2'	20.7, CH_3	2.06, s	C-9, 1'	20.9, CH_3	2.06, s	21.1, CH_3	2.06, s

Table S2. Sequences of the primers used for qRT-PCR.

Gene	Name	Primer
16S rRNA	A component of ribosomes	Forward 5'-TGT TTG ACG ATG TTT GAG CA-3'<br>Reverse 5'-CCT TCC TCC AGT TCA GAT GC -3'
agrA	Quorum-sensing regulator A	Forward 5'-TGA TAA TCC TTA TGA GGT GCT T-3'<br>Reverse 5'-CAC TGT GAC TCG TAA CGA AAA-3'
arlR	Response regulator	Forward 5'-TTA CGG TGC AGG CGA TTA TAT AG-3'<br>Reverse 5'-TAC CGT TGA CAT CGA TAA TAT CC-3'
aur	Zinc metalloprotein ase aureolysin	Forward 5'-ACC GTG TGT TAA TTC GTG TGC TA-3'<br>Reverse 5'-ATG GTC GCA CAT TCA CAA GTT T-3'
hla	α-Hemolysin	Forward 5'-CGG CAC ATT TGC ACC AAT AAG GC-3'<br>Reverse 5'-GGT TTA GCC TGG CCT TCA GC-3'
icaA	Intercellular adhesion A	Forward 5′-TGA ACC GCT TGC CAT GTG-3′<br>Reverse 5′-CAC GCG TTG CTT CCA AAG A-3′
nuc1	Nuclease	Forward 5'-CAC CTG AAA CAA AGC ATC CTA A-3'<br>Reverse 5'-TAT ACG CTA AGC CAC GTC CAT-3'
RNAⅢ	Transcriptiona l regulator	Forward 5'-ATC GAC ACA GTG AAC AAA TTC AC-3'<br>Reverse 5'-CTC TAC TAG CAA ATG TTA CTC AC-3'
saeR	Response regulator	Forward 5'-GCC TTA ACT TTA GGT GCA GAT GAC TAT GTC-3'<br>Reverse 5'-CGA CAG TTG TTC AAC TGG TTG ATG ATG G-3'
sarA	Transcriptiona l regulator	Forward 5'-GAG TTG TTA TCA ATG GTC-3'<br>Reverse 5'-GTT TGC TTC AGT GAT TCG-3'
sarZ	HTH-type transcriptional regulator	Forward 5'-CCT ATA CTG GTT ACA TTG TTT TAA TGG-3'<br>Reverse 5'-TGG TGT CAG TGT TCC AGA ATC-3'
seb	Enterotoxin B	Forward 5'-TGT TCG GGT ATT TGA AGA TGG -3'<br>Reverse 5'-CGT TTC ATA AGG CGA GTT GTT-3'
sigB	RNA Polymerase sigma factor	Forward 5'-AAG TGA TTC GTA AGG ACG TCT-3'<br>Reverse 5'-TCG ATA ACT ATA ACC AAA GCC T-3'
spa	Protein A	Forward 5'-ACC AGA AAC TGG TGA AGA AAA TCC-3'<br>Reverse 5'-TAA CGC TGC ACC TAA GGC TAA TG-3'
